# Perceptions of the Health Care Workers about the Guideline Implemented for Safe Surgical Practice during COVID-19 Pandemic in a University Teaching Hospital

**DOI:** 10.1055/s-0041-1726130

**Published:** 2021-06-03

**Authors:** Subramania Iyer, Sobha Subramaniam, Krishnakumar Thankappan, Nageswara Rao, Dipu Satyapalan, Beena Ravikumar, Anu Vasudevan

**Affiliations:** 1 Department of Head and Neck Surgery, Amrita Institute of Medical Sciences, Kochi, Kerala, India; 2 Department of Plastic Surgery, Amrita Institute of Medical Sciences, Kochi, Kerala, India; 3 Department of Pulmonology, Amrita Institute of Medical Sciences, Kochi, Kerala, India; 4 Department of Infection Control, Amrita Institute of Medical Sciences, Kochi, Kerala, India; 5 Department of Medical Administration, Amrita Institute of Medical Sciences, Kochi, Kerala, India; 6 Department of Biostatistics, Amrita Institute of Medical Sciences, Kochi, Kerala, India

**Keywords:** COVID-19 pandemic, guidelines, surgery

## Abstract

One area of health care delivery that has been affected badly in most of the institutions is the running of the surgical services. This is due to various factors such as the presence of asymptomatic carrier stage, increased morbidity and mortality in surgical procedures in a COVID-19 patient, and possible transmission of disease to the health care workers (HCWs). A guideline was formulated in our institution, which is a tertiary care university teaching hospital to resume the surgical activities in full. Following its implementation, a questionnaire-based study was conducted to understand the perception of the HCWs about the guidelines. The questionnaire had four domains with questions related to the impact of the epidemic on the practices, composition of the guidelines, its implementation, and effects. There were 217 responders which included doctors and the supportive staff. Majority of the responders welcomed the introduction of the guidelines, and felt that it ensured patient's safety and helped streamline the services. Quarantine and preoperative reverse transcription polymerase chain reaction testing were found to be appropriate measures by the respondents. In some areas, there was a difference in the responses from the doctors to that from the supportive group which assumed statistical significance. These included the reason for drop in patient numbers was the reduced patient accessibility which was felt mainly by the doctors. The doctors perceived a delay in carrying out the work, increased workload, and mental agony due to the presence of the guidelines.

COVID-19 pandemic, apart from its direct effect on health due to the viral infection, has affected the health status of many individuals by its impact on the health delivery system. This impact has been mainly due to relocation of health resources for treating the large number of existing or anticipated COVID-19 patients when extensive community spread of the disease is present. The fear of spread of the disease to the health care workers (HCWs) and other patients has made medical institutions to suspend most of the medical and surgical services. One area of health care delivery system that has been affected badly in most of the institutions is the provision of surgical services. Elective surgery was suggested to be curtailed as per most of the government advisories and guidelines issued by various national surgical societies. The effect of the delay or denial of surgical care for cancer patients as well as other ailments will definitely affect overall cure rate as well as quality of life of many patients. Resumption of these services was essential. But factors such as the presence of asymptomatic carrier stage, rapid infectivity, increased morbidity and mortality in surgical procedures when performed in a COVID-19 patient, and transmission with increased viral load during surgery to the HCWs if the patient is COVID-19 carrier make this resumption difficult and worrisome to the entire workforce of the hospital. Hence, a guideline was formulated in our institution, which is a tertiary care university teaching hospital with average 1,200 surgical major procedures every month. This was prepared based on the existing published articles 1 2 3 4 5 6 7 8 9 and advisories from health agencies. They were developed by the senior surgical and anesthetist's team with inputs from the medical administrators, the infection control department, and the senior nursing staff manning the operation rooms. These guidelines helped us resume the services soon after the national lockdown in India which was declared on March 23, 2020. Feedbacks were essential to address lacunae in the structure and implementation of the guidelines. With this aim, a questionnaire was prepared and distributed among the HCWs from the surgical departments to assess the effectiveness of the guidelines. This article reports the findings of the survey and analyses the positive and adverse responses from medical and supportive staffs.

## Brief Outline of the Guidelines Implemented

**Fig. 1:**
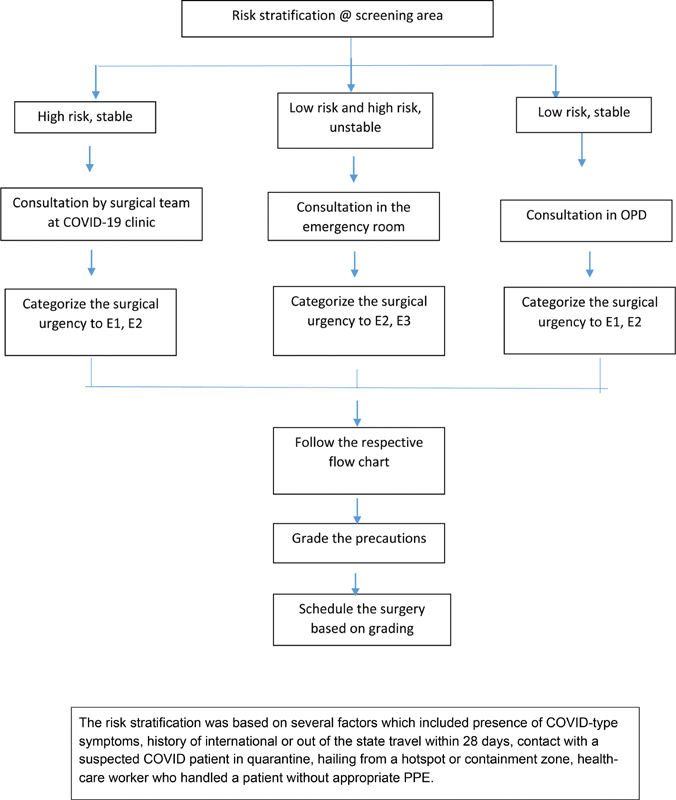
Patient pathway for scheduling the surgery. The risk stratification was based on several factors which included the presence of COVID-19–type symptoms, history of international or out of the state travel within 28 days, contact with a suspected COVID-19 patient in quarantine, hailing from a hotspot or containment zone, health care workers who handled a patient without appropriate personal protective equipment.

**Fig. 2:**
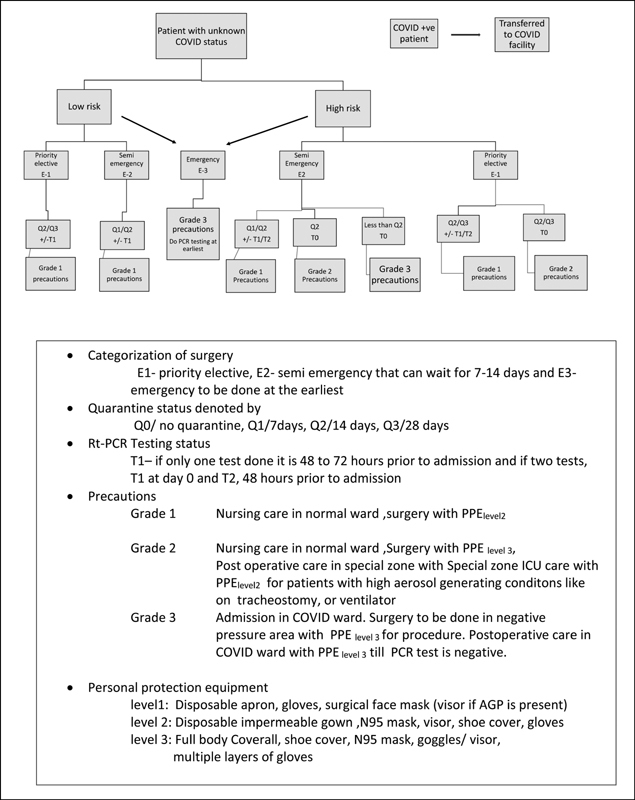
Categorization of surgery: E1, priority elective; E2, semiemergency that can wait for 7 to 14 days; and E3, emergency to be done at the earliest. Quarantine status denoted by: Q0/no quarantine, Q1/7 days, Q2/14 days, Q3/28 days. RT-PCR testing status: T1 if only one test done, it is 48 to 72 hours prior to admission and if two tests, T1 at day 0 and T2 at 48 hours prior to admission. Precautions: Grade 1 nursing care in normal ward, surgery with PPE _level 2_ . Grade 2 nursing care in normal ward, surgery with PPE _level 3_ , postoperative care in special zone with special zone ICU care with PPE _level 2_ for patients with high aerosol generating conditions such as on tracheostomy or ventilator. Grade 3 admission in COVID ward. Surgery to be done in negative pressure area with PPE _level 3_ for procedure. Postoperative care in COVID ward with PPE _level 3_ till PCR test is negative. Personal protection equipment: level 1: disposable apron, gloves, surgical face mask (visor if AGP is present). Level 2: disposable impermeable gown, N95 mask, visor, shoe cover, and gloves. Level 3: full body coverall, shoe cover, N95 mask, goggles/visor, and multiple layers of gloves. AGP, aerosol generating procedure; ICU, intensive care unit; PCR, polymerase chain reaction; PPE, personal protective equipment; RT, reverse transcription.

The guidelines assumed that when community spread is present or declared, each patient should be considered to have the possibility of harboring the virus. The guidelines were formulated on the information available from published literature, guidelines issued by various professional bodies and the governmental agencies. The guideline triaged the patient based on the urgency of the surgery and whether the patient belonged to COVID-19 high- or low-risk groups. The precautionary measures included usage of various types of personal protective equipment (PPE), quarantine, and preoperative polymerase chain reaction (RT-PCR) testing. The guidelines prescribed three grades of precautions based on the risk stratification and also the norms for preoperative and postoperative ward care, and patient transfer protocols. The flow chart based on these guidelines is depicted in Figs. 1 and 2 . The guidelines got prepared at the start of the first lockdown (March 23, 2020) in India. But full implementation started by the second lockdown period and is continuing till this report is prepared, with timely modifications based on changing prevalence, governmental regulations, and results of this survey.

## Methods

**Fig. 3:**
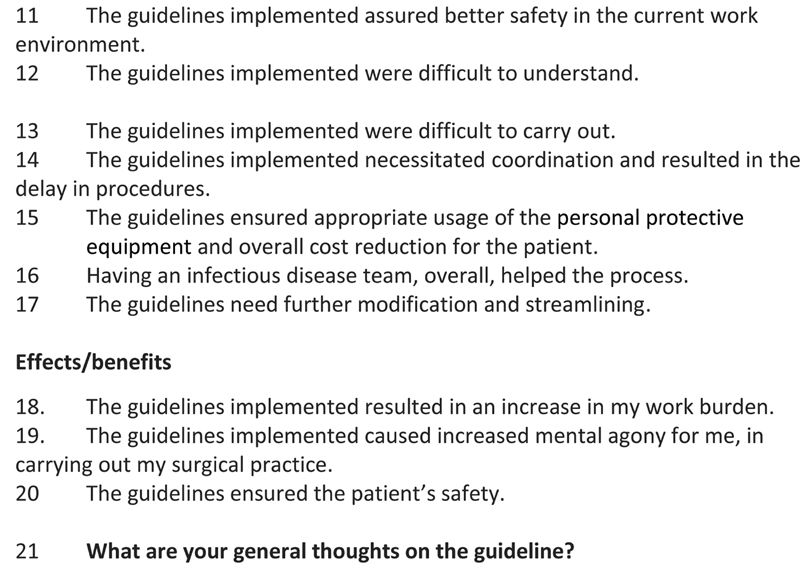
Questionnaire.

The questionnaire was prepared and the responses were collected, ∼2 weeks after the full implementation of the final version of the guidelines. The questionnaire ( Fig. 3 ) had four domains. The first was regarding the impact of the epidemic on the practices; the second domain had questions related to the formulation of the guidelines followed by set of questions to look at its implementation (third domain) and effects (fourth domain). Finally, free hand column was left for suggestions for improvement. Both English and Malayalam language versions were created. Even though linguistic validation was not performed, the translation as well as back translation was done by experts. This was circulated among the different categories of HCWs. These included surgical consultants, surgical residents, anesthesia consultants and residents, nursing staff, and paramedical staff including technicians and the administrative personnel. Personnel working in all surgical specialties were included. The questions were sent via Google Docs link and were to be answered online with anonymity being maintained. Signing in with own e-mail ID was essential to make sure that duplications do not occur, but software had the inbuilt ability to maintain the anonymity.

**Figure:**
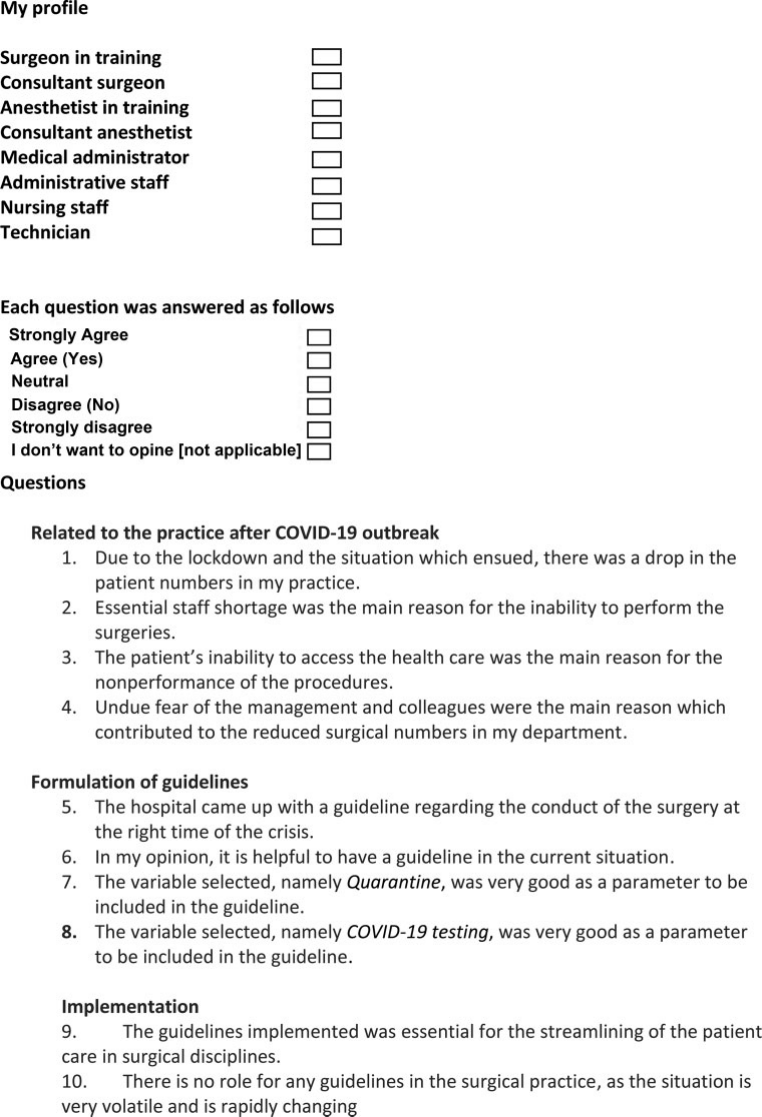


### Statistical Analysis

Analysis was done using IBM SPSS version 20 (SPSS Inc., Chicago, IL). The results which are given as percentage with 95% confidence limit were used for all categorical variables. To obtain the association of categorical variables, chi-square test was applied. A *p* -value of < 0.05 was considered as statistically significant

## Results

**Fig. 4:**
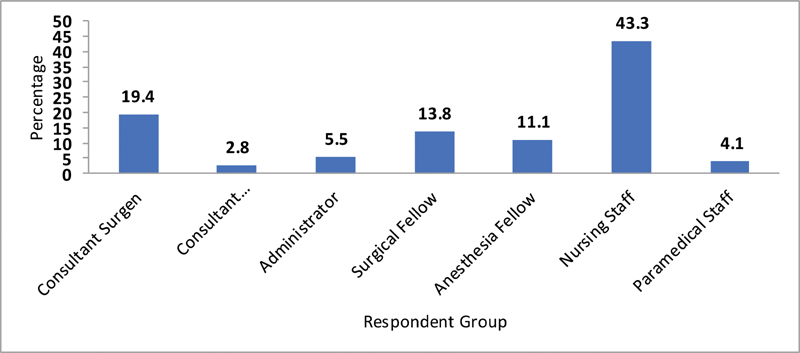
Frequency of responders.

Out of the total 217 respondents, nursing staff constituted 43.3%, surgeons 19.4%, surgical residents 13.8%, anesthetists 13.9%, technicians 4.1%, and administrative staff 5.5% ( Fig. 4 ).

### Questions Related to the Practice

**Fig. 5:**
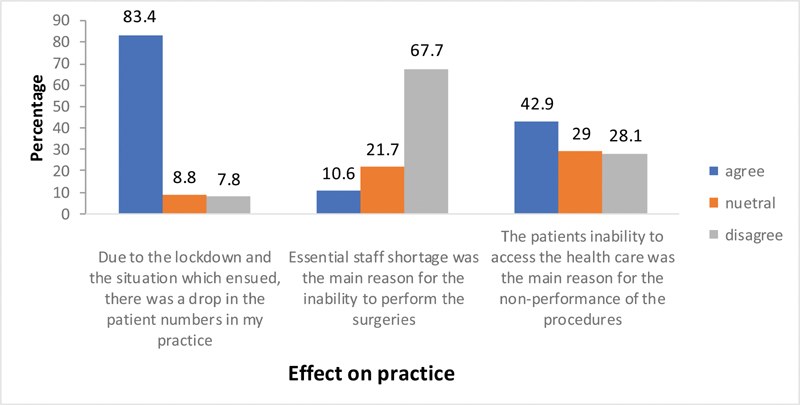
Effect of COVID-19 on practice.

Large majority of respondents (83.4%) agreed that there was a significant drop in the number of patients. Majority were of the opinion that it was not due to the shortage of staff (67%) or due to undue fear of colleagues about the disease (81%). Forty-one per cent attributed the drop in the number of patients, due to the logistic difficulties they faced in accessing the health care facilities ( Fig. 5 ).

### Questions Related to the Formulation of Guidelines

**Fig. 6:**
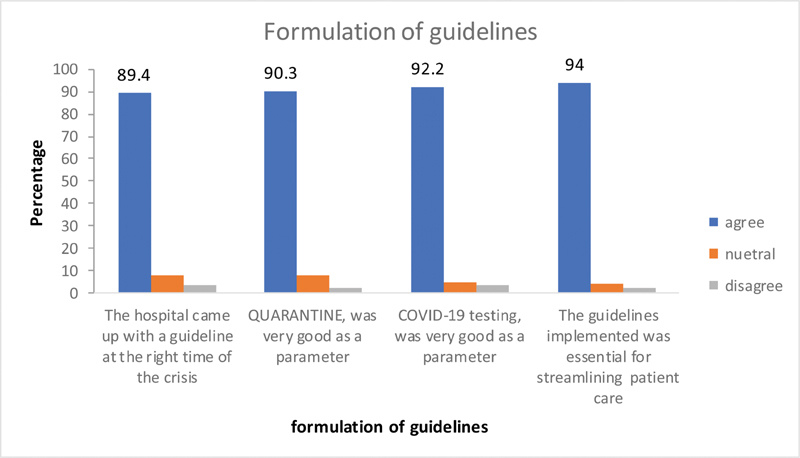
Questions related to formulation of guidelines.

The vast majority (90%) felt that the institution came up with the guidelines at the right time and almost all (99%) agreed that the guidelines were helpful and 94% felt it was essential. Regarding the variables selected in formulating the triage principles for ensuring safety, the “quarantine” and the “COVID RT-PCR testing” were found to be appropriate by 90 and 93% respondents, respectively ( Fig. 6 ).

### Questions Related to the Implementation

**Fig. 7:**
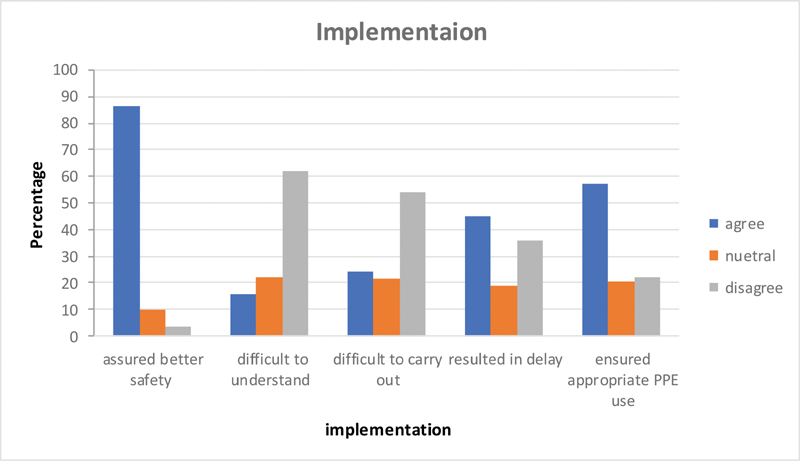
Questions related to implementation.

Majority (86%) felt that the guidelines implemented assured safety in the work practices and 89% felt the presence of an infection control department overseeing these to be helpful. On the question whether the guidelines were difficult to understand and carry out, there was a mixed response with ∼60% disagreeing and 20% agreeing with others being neutral; 45% of respondents felt that the guidelines caused delay in the procedures due to the excessive coordination needed. Fifty-seven per cent felt that the guidelines helped the appropriate usage of personal protection kits and overall cost reduction ( Fig. 7 ).

### Questions Related to the Effects or Benefits

**Fig. 8:**
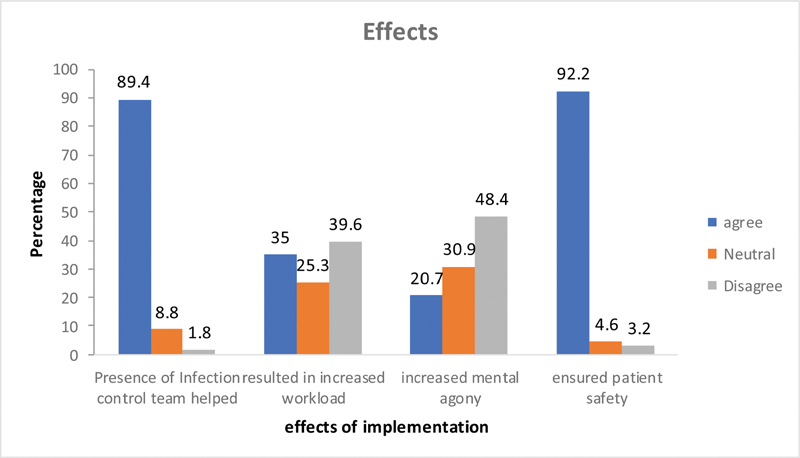
Questions related to effects of implementation.

Forty per cent of the respondents felt that the guidelines increased the work burden. To the specific question that whether the guidelines caused mental agony, only 20% felt so. A very large majority (93%) felt that the guidelines ensured patient safety and also that the guidelines would need modifications and streamlining as the situation evolves. The presence of an infection control department to advise in COVID-19 precautions was appreciated by 89% of the responders ( Fig. 8 ).

**Fig. 9:**
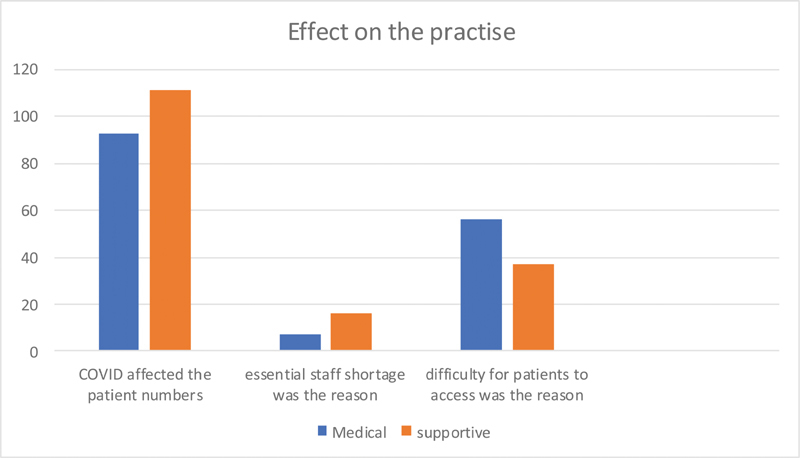
Effect on practice as felt by medical and supportive staffs.

In majority of the questions, there was a near unanimity among all the responders in their response. But in few areas, the response was divided in nature. These were analyzed to see whether there was any difference among the medical (doctor) versus supportive (nurse, technician, and administrator) groups. Among the reasons for drop in number of the patients during the COVID-19 time, difficulty felt by the patients to have access to the hospital was thought to be a reason by medical group and not by the supportive staff with a statistically significant difference ( Fig. 9 ).

**Fig. 10:**
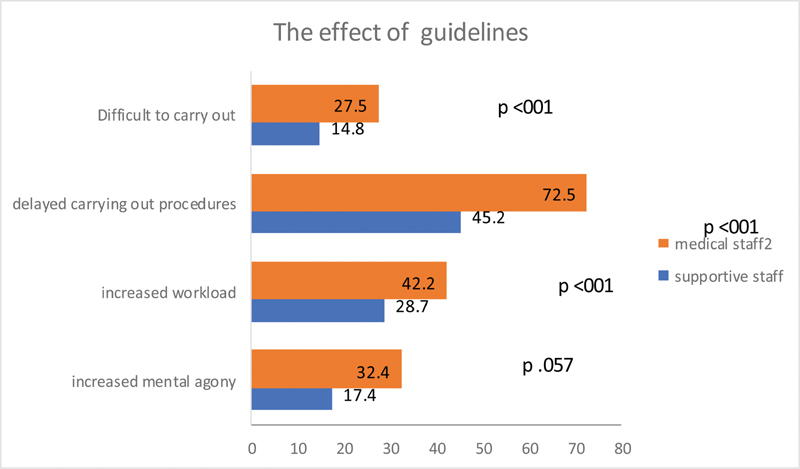
Effect of guidelines differences between medical and supportive staffs.

The medical group expressed the view that the guidelines were difficult to carry out as compared with the view by the supportive group ( *p* < 0.001). The medical group also felt that the guidelines delayed carrying out of the procedures ( *p* < 0.001). More number of medical staff felt that the guidelines increased the work burden ( *p* < 0.001). To the question whether the guidelines increased mental agony, there was more agreement from the medical group than the supportive group ( *p* < 0.052) ( Fig. 10 ).

## Discussion

The COVID-19 pandemic has created great disruption in the delivery of health services to the non-COVID-19 population all over the world. The disruption has been felt markedly in the running of surgical services with the fear of increased mortality and increased disease spread being the commonly cited reasons. The nature of the virus transmission and rapid spread coupled with the health advisories created a sense of insecurity among the HCWs, health administrators, as well as the patients and their families. Steps had to be put in place to allow safe surgical practices at the earliest. Even though the surgical societies across the world had put up their own guidelines, they did not help the day-to-day running of the service. They were mostly concerned with the type of procedure to be chosen and the modifications in the conduct of surgery and anesthesia to be adopted. Hence, the present guidelines and surgical workflow were created and implemented. These suggested a clear workflow for patients who were categorized as per their urgency of treatment, the risk of COVID-19 positivity, as well as the precautionary measures to be adopted at each level. We could continue provision of the services to a great level implementing these guidelines. Being a dynamic situation, the guidelines need to be adaptive to the changing situation. This necessitated feedback from all those who were responsible in running the surgical services. Hence, this questionnaire was prepared.

There was an obvious fall in the number of patients attending the clinics. The majority of the respondents especially the doctor group thought that this was due to the logistic difficulties the patients had in accessing the hospital facilities. The lockdown and other restrictions in the transportation may be the reason for this. This will need to be addressed by facilitating transportation and hospital care for non-COVID-19 patients by both governmental agencies and hospital administrators.

In general, the responses to the implementation-related questionnaire were positive. The respondents were of the opinion that the guidelines ensured work safety, helped in streamlining the patient care, and appropriate usage of PPE. Aziz et al's 10 study of rapid guidelines strongly recommended that each state/province/country develops a triage protocol and system to support legal framework to permit triage in clinical setting, which is based on local practices and legislation. The guidelines detailed the way of proper donning and doffing of PPE by the staff, minimizing the number of staff entering the patients' room, remote access to equipment controls and bundle care, develop and implement response plans to endotracheal intubation, cardiac arrest for patients with COVID-19.

Implementation of these guidelines was found to cause some delays in the delivery of treatment. Among the responders, the medical group felt more concerned about the delay. The difficulties in implementation of the guidelines were also felt more by the medical group. This may be explained by the fact that they were really taking more responsibility in getting the patients organized for the surgical treatment.

The respondent felt that the guidelines ensured patient safety. Majority of them did not appreciate any increased work burden with some disparity between the medical and supportive staffs. The proportion of those who felt having increased workload was more among the medical staff, probably due to the need of triaging, ensuring the quarantine and COVID-19 test status.

Xiao et al's 11 multicenter cross-sectional survey of psychological levels during COVID-19 pandemic showed that 55.1% of participants had psychological stress higher than during severe acute respiratory syndrome; 54.2 and 58% of HCWs had symptoms of anxiety and depression. The authors concluded that independent risk factors for anxiety and depression were gender, professional title, protective support, and contact history. Spoorthy et al 12 in a recent literature review showed that nurses had higher anxiety and depressive symptoms as compared with doctors. A scoping review by Shaukat et al 13 showed that HCWs experienced high levels of depression, anxiety, insomnia, and distress in this COVID-19 pandemic. Female HCWs and nurses were disproportionately affected. The German study of Zerbini et al 14 showed that job strain due to increased workload, organizational changes in working team and conflicts with colleagues, and uncertainty about the future due to health care system and economic crisis were the most common causes for psychosocial burden. In our questionnaire, the presence of mental agony was found to be not great, but relatively, this was more among the medical staff contrary to the previous studies. But in the present study, the psychological effects were not addressed in detail but only with a single question, hence may not be fully representative of the real picture.

The analysis of the feedback suggested that the implementation of these guidelines helped greatly to streamline the surgical activities. It improved the morale of the staff and allowed them to undertake surgical procedures with confidence. A study from our institution showed that with implementation of these guidelines, our surgical workload equaled 60% of that during a similar period during last year. 15 Based on the findings of this survey, we incorporated steps to reduce the delay in the work execution and provided more secretarial assistance to the medical staff to reduce their increased workload created by implementing the guidelines.

## Limitations of the Study

The questionnaire may reflect the attitude of staff from an academic hospital. Since the working environment may be different in other types of medical providers, the findings may not represent the entire spectrum of types of hospitals.

## Conclusion and Future Directions

The present study showed that implementation of the guidelines for ensuring safe surgical practice was welcomed by all the HCWs, which included both doctors and supportive staff. Insisting on quarantine and preoperative RT-PCR testing were found to be appropriate measures by the respondents. Compared with the supportive staff, the doctors felt that patient accessibility was a reason for drop in the number and felt more concerned about the delay and increased workload created by these guidelines. But all uniformly felt that it ensured patient safety as well as streamlined the services. The findings of the study will indicate that in future immediate steps should be taken to implement similar guidelines at the earliest if such situations arise and that these should be dynamic in nature taking into account the differing concerns of the medical and supportive staffs.

## References

[1] Day A T Sher D J Lee R C Head and neck oncology during the COVID-19 pandemic: reconsidering traditional treatment paradigms in light of new surgical and other multilevel risks Oral Oncol 2020 105 104684 32330858 10.1016/j.oraloncology.2020.104684 PMC7136871

[2] Lauer S A Grantz K H Bi Q The incubation period of coronavirus disease 2019 (COVID-19) from publicly reported confirmed cases: estimation and application Ann Intern Med 2020 172 09 577 582 32150748 10.7326/M20-0504 PMC7081172

[3] Lei S Jiang F Su W Clinical characteristics and outcomes of patients undergoing surgeries during the incubation period of COVID-19 infection. E Clin Med (Northfield Ill) 2020 23 100331 10.1016/j.eclinm.2020.100331 PMC7128617 32292899

[4] COVIDSurg Collaborative Collaborative C O Mortality and pulmonary complications in patients undergoing surgery with perioperative SARS-CoV-2 infection: an international cohort study Lancet 2020 396(10243):27 38 32479829 10.1016/S0140-6736(20)31182-X PMC7259900

[5] Tran K Cimon K Severn M Pessoa-Silva C L Conly J Aerosol generating procedures and risk of transmission of acute respiratory infections to healthcare workers: a systematic review PLoS One 2012 7 04 e35797 22563403 10.1371/journal.pone.0035797 PMC3338532

[6] Wong J Goh Q Y Tan Z Preparing for a COVID-19 pandemic: a review of operating room outbreak response measures in a large tertiary hospital in Singapore Can J Anaesth 2020 67 06 732 745 32162212 10.1007/s12630-020-01620-9 PMC7090449

[7] Chinese Society of Anesthesiology, Chinese Association of Anesthesiologists Chen X Liu Y Gong Y Perioperative management of patients infected with the novel coronavirus: recommendation from the Joint Task Force of the Chinese Society of Anesthesiology and the Chinese Association of Anesthesiologists Anesthesiology 2020 132 06 1307 1316 32195699 10.1097/ALN.0000000000003301 PMC7155907

[8] Ti L K Ang L S Foong T W Ng B SW What we do when a COVID-19 patient needs an operation: operating room preparation and guidance Can J Anaesth 2020 67 06 756 758 32144591 10.1007/s12630-020-01617-4 PMC7090746

[9] Brat G A Hersey S Chhabra K Gupta A Scott J Protecting surgical teams during the COVID-19 outbreak: a narrative review and clinical considerations Ann Surg 2020 10.1097/SLA.0000000000003926 PMC7224623 32379080

[10] Aziz S Arabi Y M Alhazzani W Managing ICU surge during the COVID-19 crisis: rapid guidelines Intensive Care Med 2020 46 07 1303 1325 32514598 10.1007/s00134-020-06092-5 PMC7276667

[11] Xiao X Zhu X Fu S Hu Y Li X Xiao J Psychological impact of healthcare workers in China during COVID-19 pneumonia epidemic: a multi-center cross-sectional survey investigation J Affect Disord 2020 274 405 410 32663970 10.1016/j.jad.2020.05.081 PMC7236675

[12] Spoorthy M S Pratapa S K Mahant S Mental health problems faced by healthcare workers due to the COVID-19 pandemic--a review Asian J Psychiatr 2020 51 102119 32339895 10.1016/j.ajp.2020.102119 PMC7175897

[13] Shaukat N Ali D M Razzak J Physical and mental health impacts of COVID-19 on healthcare workers: a scoping review Int J Emerg Med 2020 13 01 40 32689925 10.1186/s12245-020-00299-5 PMC7370263

[14] Zerbini G Ebigbo A Reicherts P Kunz M Messman H Psychosocial burden of healthcare professionals in times of COVID-19 - a survey conducted at the University Hospital Augsburg Ger Med Sci 2020 18 Doc05 32595421 10.3205/000281 PMC7314868

[15] Iyer S Subramaniam S Ravikumar B Recommendations for safely performing major head and neck surgery during the COVID-19 pandemic: experience with implementation of a workflow J Maxillofac Oral Surg 2020 19 04 1 8 32905141 10.1007/s12663-020-01444-6 PMC7466925

